# Optimal fluid management for the surgical intensive care unit patient

**DOI:** 10.62675/2965-2774.20250035

**Published:** 2025-09-22

**Authors:** Robert Wise, Prashant Nasa, Manu L. N. G. Malbrain

**Affiliations:** 1 Department of Anaesthesia and Critical Care School of Clinical Medicine University of KwaZulu-Natal Durban South Africa Department of Anaesthesia and Critical Care, School of Clinical Medicine, University of KwaZulu-Natal - Durban, South Africa.; 2 Intensive Care Department John Radcliffe Hospital Oxford University Trust Hospitals Oxford United Kingdom Intensive Care Department, John Radcliffe Hospital, Oxford University Trust Hospitals - Oxford, United Kingdom.; 3 Department of Anaesthesia and Critical Care Medicine New Cross Hospital The Royal Wolverhampton NHS trust Wolverhampton UK Department of Anaesthesia and Critical Care Medicine, New Cross Hospital, The Royal Wolverhampton NHS trust - Wolverhampton, UK.; 4 First Department of Anaesthesia and Intensive Therapy Medical University of Lublin Lublin Poland First Department of Anaesthesia and Intensive Therapy, Medical University of Lublin – Lublin, Poland.; 5 International Fluid Academy Dreef 3 Lovenjoel Belgium International Fluid Academy, Dreef 3 - Lovenjoel, Belgium.; 6 Medical Data Management Medaman Geel Belgium Medical Data Management, Medaman - Geel, Belgium.

## INTRODUCTION

Optimal fluid management in surgical intensive care unit (ICU) patients is a complicated balance between avoiding unnecessary intravenous (IV) fluid administration, which contributes to fluid accumulation syndrome (FAS), and maintaining euvolemia to promote tissue and organ perfusion.^1,2^Fluid kinetics and dynamics are influenced by the underlying pathology, shock state, administered drugs, the phase of disease, and fluid management (‘ROSE model’, acronym from resuscitation, optimisation, stabilisation, or evacuation/ recovery phase).^3^Surgical patients may often follow similar phases of fluid modelling from admission through the emergency department, the operating theatre, the ICU, and the hospital wards.

**Figure 1:**
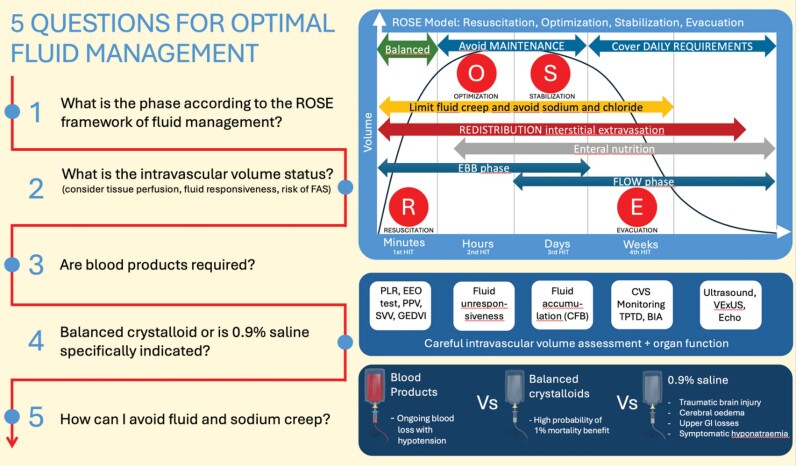
Questions for optimal fluid management. FAS - fluid accumulation syndrome; PLR - passive leg raising; EEO - end-expiratory occlusion; PPV - pulse pressure variation; SVV - stroke volume variation; GEDVI - global end-diastolic variation index; CFB - cumulative fluid balance; CVS - cardiovascular system; TPTD - transpulmonary thermodilution; BIA - bioelectrical impedance analysis; VExUS - Venous Excess Ultrasound Score; Echo – echocardiography; GI - gastrointestinal.

Fluid management is further complicated by the damaged endothelial glycocalyx layer, which usually maintains fluid in the intravascular compartment.^4^ In the absence of an intact and functioning endothelial glycocalyx layer, all fluids administered IV inevitably leaks into the interstitial space and contributes to FAS and a vicious cycle of tissue oedema and hypovolemia.^1^ This “viewpoint review” aims to provide an evidence-based perspective on optimal fluid management in surgical ICU patients. A stepwise approach to clinical scenarios in such patients may be helpful, starting with identifying the phase of fluid management (Figure 1).

### ROSE PRINCIPLE

Shocked surgical patients presenting in the resuscitation phase need timely restoration of intravascular volume through goal-directed resuscitation. This can be aided by clinical examination, intravascular volume and fluid responsiveness assessment, thromboelastography^5^ when available, especially in ongoing hemorrhage, and careful early vasopressor therapy (particularly in sepsis).^6,7^ Balanced crystalloids are an appropriate first choice; however, most studies support the early administration of a fixed balance ratio of blood products when there is significant blood loss, particularly in major trauma.^8,9^

The rationale for balanced crystalloids extends to the perioperative, when it probably improves perioperative acid-base status despite an unknown effect on mortality and need for renal replacement therapy.^10^ Further research in this area is required. Evidence supports the use of balanced crystalloid administration in adult critically ill patients in a non-neurosurgical setting.^11^

A keen awareness should remain to avoid unnecessary crystalloid fluid administration, which will later contribute to FAS^1,12^Equally, blood products should be avoided when not indicated. While unnecessarily excessive IV fluid strategies result in harm, unnecessarily deliberate restrictive fluid strategies also carry risks. The RELIEF trial did not show benefit from a restricted strategy in elective major abdominal surgery, but it did result in a higher rate of acute kidney injury.^13^ Intravenous fluid strategies intra-operatively should aim for euvolemia. However, when volume status is unknown or difficult to assess in an elective surgical population, it seems reasonable to use a modestly liberal fluid regimen instead of a restricted one, considering current evidence. Whether this applies to all surgical populations requires further research.

Optimization of intravascular volume and perfusion should continue in hypovolaemic patients until they are no longer fluid responsive or until further fluid administration poses greater risk than benefit, whichever occurs first. Critically ill patients are particularly susceptible to fluid extravasation, which causes fluid accumulation across multiple organs, such as the lungs and gastrointestinal tract, and causes intra-abdominal hypertension. Thus, IV fluid should not be given if the complications outweigh the benefits.

Fluid administration in the stabilisation phase is dominated by nutrition and medication.^12^ Evacuation of accumulated interstitial fluid usually occurs spontaneously with restoration of the glycocalyx.^4^ The evidence for the benefit of routinely using diuretics to augment interstitial fluid removal is uncertain.

### VOLUME ASSESSMENT

Frequent and careful assessment of intravascular volume is important before administering additional IV fluids, particularly boluses. There is proven utility in assessing pre-load responsiveness with correctly performed dynamic manoeuvres such as a passive leg raise test (in both mechanically ventilated [MV] and non-ventilated patients) or the end-expiratory occlusion or tidal volume variation test (in MV patients). Pulse pressure variation and stroke volume variation, together with bedside ultrasound and echocardiography, provide additional data points aiding decision-making.^14^

Assessment for hypervolaemia is as important as hypovolaemia. Beware using peripheral edema as a marker of volume status as it may exist in both intravascularly hypervolaemic and hypovolaemic patients (because of endothelial glycocalyx layer damage).^4^ As such, total cumulative fluid balance is less helpful because it does not accurately reflect intravascular volume. The Venous Excess Ultrasound Score (VExUS) score may help determine venous congestion. Lung ultrasound in combination with bio-electrical impedance analysis provides further information on the state of FAS.^1^

Static indices, such as central venous pressure, are less helpful, but extreme values prompt further clinical evaluation. Central venous pressure does offer information on right sided heart function and the possibility of pericardial tamponade or obstructive shock.

### CHOICE OF FLUID

Current evidence supports a restrictive transfusion trigger for most critically ill surgical patients. Guidelines for transfusion thresholds in traumatic brain injury do not generally support either a liberal or restrictive strategy, and a recent systematic review and frequentist-Bayesian meta-analysis could not find definitive support for a liberal strategy. However, recent trials suggest a long-term neurological outcome advantage for liberal strategies, and further research is required.^15,16^ Debate still exists for surgical oncology patients with a need for further research.

There appears to be little evidence to support synthetic colloid use in critically ill patients.^17^Large prospective studies in trauma patients are lacking. However, retrospective observational studies and meta-analyses do not show an advantage to using synthetic colloids as the volume expansion effect is similar to crystalloids in the peri-operative setting.^3,18^

We advise using balanced crystalloids in most critically ill surgical patients. While data are heterogeneous, there is a high probability (> 90%) that balanced crystalloids help reduce mortality by 1% (range -9 to +1%) when compared with 0.9% saline, especially in sepsis but also in burns and diabetic ketoacidosis.^10^ Exceptions would be cases of traumatic brain injury, cerebral oedema (where tonicity of fluid is critical), metabolic alkalosis (e.g., upper gastrointestinal losses due to vomiting), and symptomatic hyponatraemia and hypochloraemia.

There is little evidence to demonstrate the benefit of hypotonic *versus* balanced crystalloid solutions for maintenance purposes. The authors advise a balanced hypotonic fluid strategy during the stabilisation and evacuation phases. This should provide maintenance requirements to cover daily needs without additional unnecessary sodium and chloride, which could contribute to further electrolyte and metabolic issues.

### DAILY REQUIREMENTS

Once nutrition and medications are considered, IV fluids can be shifted to compositions explicitly designed for maintenance. These fluids are usually hypotonic and contain the daily electrolyte and water requirements (Table 1), thus not overloading patients with either sodium or chloride. Many patients receive enough fluids from other sources (e.g., drug dilution, medications, feed) and no longer require additional IV maintenance fluids.^3^ Replacement fluids, on the other hand, should mimic the lost fluid (e.g., isotonic balanced solutions in cases of high output ileostomies).

**Table 1:** Daily water and electrolyte requirements for adults(19)

Water	Sodium	Chloride	Potassium	Glucose
1mL/kg/hour	1.5mmol/kg/day	1mmol/kg/day	1mmol/kg/day	1 - 1.5g/kg/day

The role of exogenous albumin is unknown in most surgical ICU patients. Recent guidelines advise the routine use of albumin only for patients with cirrhosis and ascites undergoing large-volume paracentesis, hepatorenal syndrome, and those with cirrhosis and spontaneous bacterial peritonitis.^20^Further studies need to investigate the role of albumin in reducing the volume of crystalloid fluid resuscitation and subsequent risk of FAS.

### ASSESSING THE CONSEQUENCES

Most indices assessing the response to administered IV fluid target the macrovascular circulation and do not assess microvascular perfusion. Traditional surrogate perfusion markers include lactate, base deficit, central/mixed venous oxygen saturation, and venous-to-arterial CO_2_ tension difference (pCO_2_ gap). Combining these indices with clinical examination (capillary refill time and skin mottling) and haemodynamic monitoring values is frequently advocated to assess responses to IV fluid administration.^14^ Calibrated haemodynamic monitoring should be applied when available in cases of vasopressor therapy use, worsening shock, or cardiac dysfunction is suspected.^14^Results from the ANDROMEDA-SHOCK-2 trial are awaited to identify if hemodynamic phenotype-based, capillary refill time-targeted resuscitation offers a superior strategy for resuscitation in septic shock, and whether this applies to surgical ICU patients.

### OTHER CONSIDERATIONS

The unintentional administration of excessive fluids and sodium loading is prevalent in ICUs and occurs mostly through maintenance and replacement fluids.^12^ Van Regenmortel et al. showed how fluid and sodium creep are responsible for a third of the mean daily fluid volume. It is vital to consider all fluid intake when deciding on fluid strategies, including feeds and medications. Equally, electrolyte loading in fluids, feeds, and medications needs consideration to avoid hypernatremia and hyperchloremia.

## CONCLUSION

Optimal fluid management in surgical intensive care unit patients requires careful and frequent assessment of volume status, an understanding of the ROSE phases of fluid management, indications for blood transfusion, and a focus on achieving adequate perfusion with balanced crystalloids in most instances of resuscitation. Maintenance fluids should include feeds and medications before using hypotonic maintenance fluids designed for this purpose. Unnecessary fluid administration needs to be actively avoided.
